# *Legionella Pneumophila* and Dendrimers-Mediated Antisense Therapy

**DOI:** 10.15171/apb.2017.022

**Published:** 2017-06-30

**Authors:** Roghiyeh Pashaei-Asl, Khodadad Khodadadi, Fatima Pashaei-Asl, Gholamreza Haqshenas, Nasser Ahmadian, Maryam Pashaiasl, Reza Hajihosseini Baghdadabadi

**Affiliations:** ^1^Department of Biology, Payame Noor University, Tehran, Iran.; ^2^Department of Anatomy, Medical School, Iran University of Medical Science, Tehran, Iran.; ^3^Cellular and Molecular Research Center, Iran University of Medical Sciences, Tehran, Iran.; ^4^Genetic Theme, Murdoch Children's Research Institute, Royal Children's Hospital, The University of Melbourne, Melbourne, Australia.; ^5^Molecular Biology Laboratory, Biotechnology Research Center, Tabriz University of Medical Sciences, Tabriz, Iran.; ^6^Microbiology Department, Biomedical Discovery Institute, Monash University, Melbourne, Australia.; ^7^Transplantation Center, Department of Curative Affairs, Ministry of Health and Medical Education, Tehran, Iran.; ^8^Drug Applied Research Center, Tabriz University of Medical Sciences, Tabriz, Iran.; ^9^Department of Anatomical Sciences, Faculty of Medicine, Tabriz University of Medical Sciences, Tabriz, Iran.

**Keywords:** Legionella pneumophila, DrrA (SidM), LepA and LepB, Intracellular replication, Rab, Vesicular trafficking

## Abstract

Finding novel and effective antibiotics for treatment of *Legionella disease* is a challenging field. Treatment with antibiotics usually cures *Legionella* infection; however, if the resultant disease is not timely recognized and treated properly, it leads to poor prognosis and high case fatality rate. *Legionella pneumophila* DrrA protein (Defects in Rab1 recruitment protein A)/also known as SidM affects host cell vesicular trafficking through modification of the activity of cellular small guanosine triphosphatase )GTPase( Rab (Ras-related in brain) function which facilitates intracellular bacterial replication within a supporter vacuole. Also, *Legionella pneumophila* LepA and LepB (Legionella effector protein A and B) proteins suppress host-cell Rab1 protein’s function resulting in the cell lysis and release of bacteria that subsequently infect neighbour cells. Legionella readily develops resistant to antibiotics and, therefore, new drugs with different modes of action and therapeutic strategic approaches are urgently required among antimicrobial drug therapies;gene therapy is a novel approach for *Legionnaires disease* treatment. On the contrary to the conventional treatment approaches that target bacterial proteins, new treatment interventions target DNA (Deoxyribonucleic acid), RNA (Ribonucleic acid) species, and different protein families or macromolecular complexes of these components. The above approaches can overcome the problems in therapy of Legionella infections caused by antibiotics resistance pathogens. Targeting Legionella genes involved in manipulating cellular vesicular trafficking using a dendrimer-mediated antisense therapy is a promising approach to inhibit bacterial replication within the target cells.

## Introduction

Legionnaires' disease is a serious form of pneumonia and lung inflammation, which is caused by intracellular bacterium Legionella. Although early therapeutic intervention using antibiotics usually cures Legionnaires’ disease, some patients experience complications that could lead to death.^1^ Legionella rapidly develops resistance to commonly used antibacterial agents.^2^ Therefore, there is an urgent demand for discovery of new antibacterial targets to overcome the resistance problem. Bacterial pathogens deliver effector proteins which interfere with host cell physiological functions and hijack their target cell machinery leading to specific clinical symptoms.^3,4^ To escape degradation by its host cells, a Legionella-containing vacuole (LCV)‏ is formed and protects the bacterium from cell immune defense, possibly through secretion of bacterial proteins into the host cytosol.^5^ Therefore, specific antibiotics with high levels of permeability are required to pass cell membrane barrier and reach the bacterium within the cells.^6^‏ This review describes different gene therapy approaches including antisense therapy mediated by dendrimers to target and eliminate or disarm pathogen, novel method for specific targeting of effective types of antibiotics to intracellular‏ *L. pneumophila (Legionella pneumophila).* We also describe antisense therapy for *L. pneumophila* treatment targeting bacterial protein synthesis aiming to disturb host trafficking pathway through interference with phagosome and lysosome fusion in macrophages, therefore targeting bacteria in the cytoplasm by different methods such as RNA interference type would be an alternative option to prevent bacterial growth and prevent the clinical symptoms.

### *Legionella pneumophila* a medically important intracellular pathogen

The most recent major outbreak of *L. pneumophila*, or Legionnaires' disease happened in Portugal in 2014, and was referred to one of the biggest in European history. If this relatively rare infection is not timely recognized and properly treated, it can have poor prognosis and present a high case fatality rate.^7^

*L. pneumophila* is a gram-negative intracellular bacterium^8^ that causes Legionnairesis in humans. The natural host of *L*. pneumophila is unicellular protozoa and it infects human alveolar macrophages.^9,10^ Despite progress in the antimicrobial treatment, pneumonia is still one of the important infectious diseases, causing death in the developed countries.^11-13^* L. pneumophila* co-infection with influenza could lead to influenza infection with possibly lethal prognosis.^14^

Following inhalation, *L. pneumophila* infects and replicates in alveolar macrophages, which contribute to inflammation and progress of the disease. *L .pneumophila*, with ability to deliver above 300 proteins to the host cell through its Type IVB, Icm/Dot (the intracellular multiplication/defective organelle trafficking) translocation system conserves the major recognized set of translocated substrates between all bacterial pathogens.^15^ Inside host cells, *L. pneumophila* avoids phagosome-lysosome fusion and influences host cell procedures to form a particular phagosome which is proper for intracellular replication.^3,8,16^ The Icm/Dot system is used by the bacterium to translocate its effectors.^4,15^

## Legionella pathogenesis

The lungs are the main site of infection, where bacteria grow inside the lung macrophages.^16-19^ In the extra pulmonary forms of disease, organs such as heart, CNS, liver and intestines are involved and heart is the most common organ involved in the hospitalized patient. Recipients of organ transplant and patients with diabetes mellitus and chronic lung disease as well as aged people and cigarette smokers are suitable candidate for this disease.^20^

### *L. pneumophila* Life Cycle 

Intracellular pathogens use different mechanisms to manipulate the host cell system for intracellular replication. For example, *L. pneumophila* as an intracellular bacterial pathogen hijacks host vesicle trafficking pathway to stop phagosome and lysosome fusion inside the cell.^4,21-23^ Lysosomes are intracellular organelles with acidic pH in eukaryotic cells containing hydrolytic enzymes for digesting cellular waste products, bacteria and viruses. Normally when bacteria enter to cell by phagocytes, they are killed in lysosomes through digesting by the lysosomal enzymes.^23^

**Figure 1:**
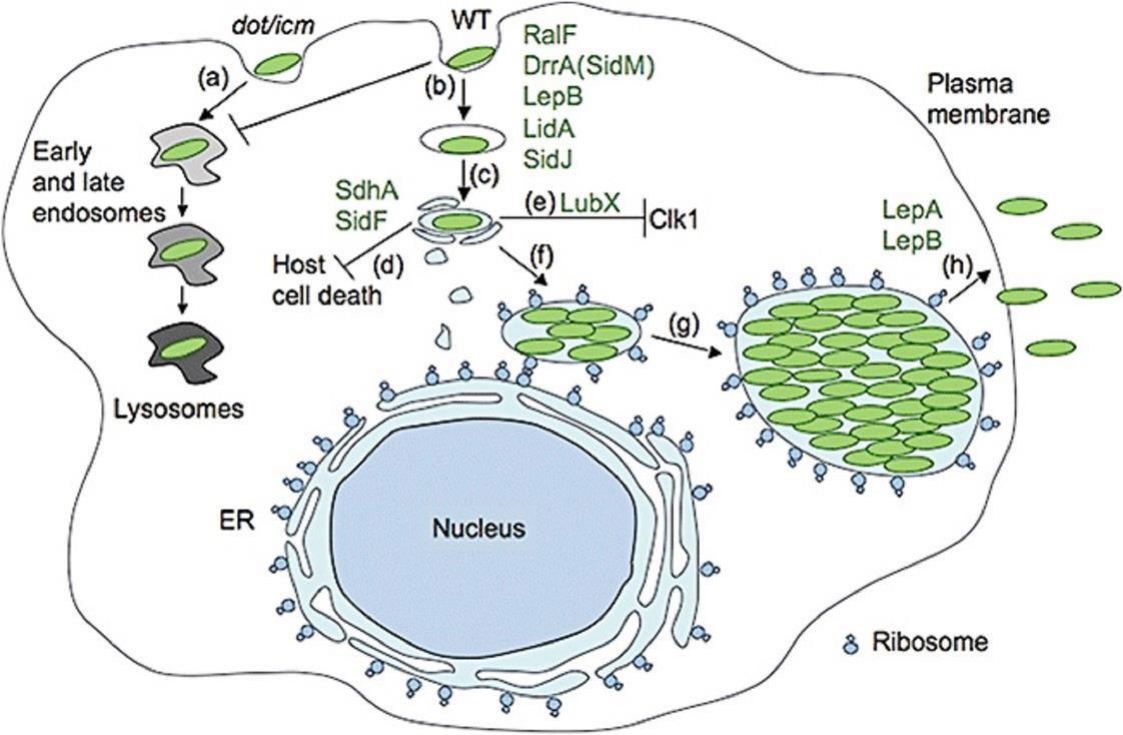
The replication of *Legionella pneumophila* inside the cell (Figure adapted from ^23^**)**
Wild-type and mutant form of Legionella use different ways for trafficking pathways(a) *dot/icm* mutants and trafficking pathway, (b) Wild-type of *Legionella Pneumophila,* the LCV formed and avoids endosomal fusion, (c) The Dot/Icm different effectors, (d) Host cell is prevented from death by the Dot/Icm effectors SdhA and Sid F, (e) the Dot/Icm effecter LubX effect the host cell factor Clk1, (f) the LCV was surrounded with Ribosomes, (g) Several cycle of *Legionella Pneumophila* replication,(h) *Legionella Pneumophila* Dot/Icm effectors LepA and B cause to *Legionella pneumophila* infect the other neighbour host cell Dot/Icm-translocated effectors is shown in green.^23^

Host cells use the defense mechanism for limiting the intracellular infection.^23^ Following cell infection by *L .pneumophila*, some immune system cells such as macrophages surround the bacteria but bacteria manipulate host cells using membrane trafficking pathway. Inside the macrophages, pathogen utilizes the host cell proteins mediating intracellular trafficking pathway. This forms an organelle termed *L. pneumophila* - containing vacuole (LCV) which supports bacteria replication (Figure 1).^9,16,21,23,24^

Type IV secretion system of *L. pneumophila* which is encoded by the Icm/Dot genes enables bacteria to transfer its proteins into host cytosol.^25-27^ A number of different translocated substrates of Icm/Dot have been identified with similar functions to eukaryotic host cell proteins involving in vesicle trafficking pathway (Figure 1).^9,23,27-31^

Rab GTPase‏ proteins in eukaryotic cells act as molecular switches and are important in cellular trafficking pathway. Following pathogen phagocytosis or endocytosis, host cell Rab GTPase proteins are essential for intracellular transportation. Bacterial effectors hijack Rab proteins at the molecular level act to escape degradation, be carried directly to specific intracellular locations, and control host vesicles carrying molecules requiring for a stable niche and/or bacterial development and differentiation.^4,32,33^

## Development of antibacterial resistance by Legionella

The discovery and therapeutic use of antibiotics in the 1950s have certainly contributed to the one of the ultimate profits to human; however, because of the short life cycle and capacity to adjust rapidly to variations in the environmental condition, pathogenic bacteria continue to persevere by regularly overcoming the effect of drugs used to eliminate them. The growing drug resistance was the first problem resulting from the extensive, uncontrolled and inappropriate use of antibiotics. In spite of the entrance of new antibiotic into the market, drug resistance is detected in years or even months. At present, more than 70% of pathogenic bacteria are resistant to most antibiotics existing on the market and the mortality of some multi-resistant infections has extended to 50 - 80% and also the mortality rate as a result of bacterial infections is above 2 millions per year, worldwide.^34,35^ Furthermore,‏ nowadays, some environmental bacterial pathogens such as Legionella spp. as a result of artificial ecosystems are a main problematic issue in industrialized countries, associated with Other factors such as modern medications and lifestyles have been caused an increased incidence of unintended pathogens in the form of emerging pathogens.^36^

According to the different approaches especially bacterial resistance and action of antibacterial medications presently used, different targets such as cellular structures, the cell wall biosynthesis, protein biosynthesis, DNA, different RNA families, biosynthetic pathway, new protein families or macromolecular complexes of these components have been suggested by commercial antibacterial companies and scientists.^35,37^ Therefore, targeting specific bacteria such as *L. pneumophila* which may present as Legionnaires’ diseases and cause case fatality rate of about 10% and even mortality rate higher than 25% in immune suppressed and nosocomial patients^11^ requires to be paid more attention.

## Targeting *Legionella* proteins by antibacterial

Finding new and effective antibiotics is a challenging research area driven by novel approaches required to tackle unconventional targets. Intracellular grown Legionella is extremely resistant to antibiotics.^38,39^ Although combination antibiotic therapy might be a choice in some conditions, it is not recommended for all patients and is a controversial and challenging issue.^40^*L. pneumophila* is able to manipulate vesicular trafficking by modification the activity of the small GTPase Rab1. *L .pneumophila* manipulates Rab1 function using some of its associate proteins such as DrrA (SidM). DrrA (SidM) has both guanine nucleotide exchange activity in Rab1 GDF (GDI dissociation factor) and Rab1 GEF (guanine nucleotide exchange factor).^19,41-43^ Also there is another protein of *L. pneumophila* named LepB which manipulate and inactivate host-cell Rab1 protein’s function.^33,41^ Following growing inside the LCV, a bacterium lyses the host cell and release to infect the neighbour cells‏. Two effectors, LepA and LepB, which show a role in the non-lytic release of Legionella from protozoa, are translocated by the Icm/Dot TFSS (Type Four Secretion System).^42,44^ Reduction of the Lep proteins through deletion of their genes contributes to better ability to lyse red blood cells. In contrast, overexpression of Lep-containing hybrid proteins seems to exactly block the activity of the Icm/Dot TFSS and may stop the transfer of other effectors which are critical for intracellular multiplication.^33,42^ Therefore, Legionella’s effectors which hijack host protein to escape degradation and replicate intracellularly could be targeted in antibacterial treatment. In addition, the LepA and the LepB proteins in Legionella are the other targets to induce infection the neighbour cells in host cells.

## Gene therapy approach for treatment of Legionella infection

Antibiotic resistance is a health threat, worldwide. In spite of good progresses in genome sequencing and genetic manipulation tools, there are still problems to be used for effective therapeutic aims.^45^ Gene therapy is a technique which causes insertion, silencing or alteration of genes in a patient's cells to treat or prevent disease.^46^ RNA regulators are developed to overcome various restrictions of protein regulators such as simple structures and mechanisms causing their behaviour in different conditions anticipated with software tools. Also they propagate signals directly and fast as RNAs.^47^ The remodeling of RNA and DNA molecules with the aim of engineering antibiotic bunches to cause antibiotic overexpression is possible.^48^ Recently, combination of CRISPR (Clusters regularly interspaced short palindromic repeats) and antisense RNA system in order to control bacterial gene expression is introduced.^47^ Through blocking genes that manipulate and inactivate host-cell Rab1 protein’s function, Legionella can’t form LCV to support bacteria inside LCV for replication.

## CRISPR-Cas system: a novel tool for treatment of Legionellosis

Not only CRISPR-Cas component is important in the natural history and pathogenesis of Legionnaires’ disease, but also *L. pneumophila Cas2* has a role that is unique from the main view of CRISPR-Cas function.^49^ CRISPR , which were first discovered in bacteria in 1980s and then in archaea in 1990s, are a powerful genome editing tool. CRISPR function to facilitate adaptation of the organism to extreme environmental conditions and act as a part of bacterial immune system to defend against pathogens and harmful environment.^50,51^ CRISPR/Cas systems are powerful and efficient genome modifying tool in comparison to other genome modifying tools like zinc-finger nucleases (ZFNs) and transcription activator-like effector nucleases (TALENs). The system includes a nuclease (Cas) and small guide RNA that recruits the Cas to cut at a specific place in the genome. This system is able to induce targeted specific gene deletion, correction or mutation via RNA guided DNA cleavage. Short palindromic repeat 36 base pair (bp) lengths in the genome associated with the (Cas) gene carry out targeted editing in the proposed genome. It works by binding a RNA stem-loop structure when attached to a short target sequence (22-33bp) to guide the Cas protein to a specific spot in the genome. These adaptable sequences together with non-contiguous direct repeat attached to Cas gene forming the CRISPR Cas systems.^52-57^

CRISPR type II, based on Cas9 is the primary system used to genetically modify mammalian cells. Cas9 function in CRISPR system is central part of the tool and is guided to the target sequence by a trans activating crRNA (tracrRNA) to cleave target DNA – Cas9 cleaves supercoiled, relaxed and linear DNA – cleavage occurs 3bp upstream of Pam motif.^54^ This type of gene editing technology has been independently described by several groups and is termed RNA guided engineered nucleases (RGENs).^58^

**Figure 2:**
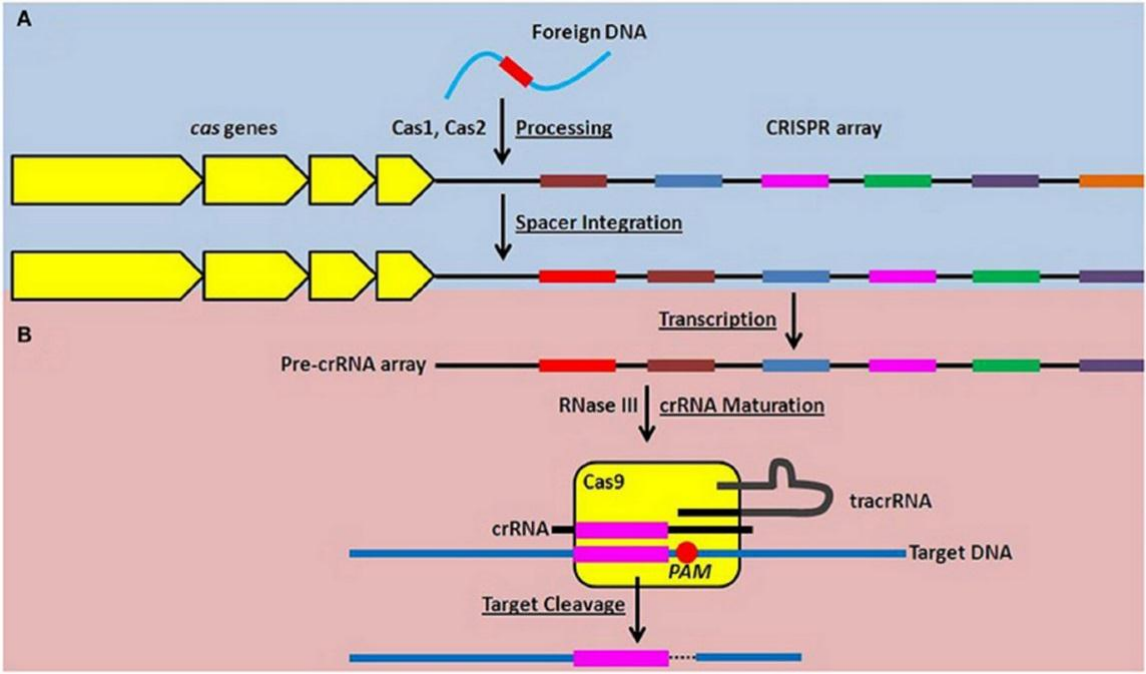
The Type II CRISPR-Cas system function in adaptive nucleic acid restriction (Figure adapted from^59^**)**
**(A**) Invading DNA is located by Cas1 and Cas2 and take it as new spacer sequence inside CRISPR array (immune completion blue). **(B)** To stop invading DNA, the pro-crRNA in constructed and matured into small targeting crRNAs to associate with Cas9 and the sequence of spacer within the crRNA can hybridize the matching DNA. Cas9 then cleavages target DNA just 3bp downstream of PAM site and generates double stranded break on target DNA.^59^

CRISPR technology has some benefits over early methods of gene editing technology and is rapidly expanding in the area of genetic and biology research. It is a relatively rapid and cost effective genome editing technology that can be used to modify the genome in different organisms and various cell types. CRISPR Cas system target the bacterial lipoproteins transcript through dual RNA protein complexes (Figure 2).^59^

## Antisense therapy of Legionellosis

Gene expression could be down regulated by RNA interference and antisense oligonucleotides (AS-ODNs) through inducing enzyme-dependent degradation of targeted mRNA**.**^60^ For treatment of different gene-specific diseases, oligonucleotide-based cure is a novel area of medicine to design new drugs. The antisense oligonucleotides and short interfering RNAs (siRNAs) are the more common forms, which often act against similar targets.^61^ Using antisense and other gene silencing technologies provide an efficient alternative way of treatment for cancer, genetic disorders or infection.^62,63^ The gene expression through changing mRNA splicing, arresting mRNA translation and inducing degradation of targeted mRNA are blocked through sequence-specific antisense oligonucleotides by RNase H.^64^ Antisense therapy is also convenient way of genetic alteration compare to more difficult methods such as generating gene knockout in cells and organisms. Antisense ODNs have been applied to efficiently block gene expression in eukaryotic cells and there is one antisense ODN-based product in the market and others in clinical trials.^65,66^ This gene therapy method is common for silencing the abnormal gene to stop the human disease such as cancer progression^67-69^ and neurodegenerative disorders^70^ as well as pneumonia.^71^

Antisense transcription was first revealed in bacteria about 50 years ago. The significant amount of antisense transcription is an important feature of gene expression in eukaryotes. This technique mostly uses DNA or RNA to inactivate circular segment of bacterial genome resembling RNA interference and prevents duplication of bacterial cells or kills them;^62,71^ however, antisense technology is not applied widely in prokaryotic systems. Gene regulations in prokaryotic cells have been done by antisense mechanisms of bacteria and also through increased antibiotic efficacy.^35,45,62,72^ Former reports indicated short antisense and modified antisense oligodeoxynucleotide (AODNs) could inhibit gene expression in bacteria.^45,72,73^ Others stated that gene expression in bacteria can be inhibited by peptide nucleic acid (PNA).^71,74^ The transcriptomes of bacteria such as Escherichia coli, Synechocystis sp. strain PCC6803, Helicobacter pylori, Bacillus subtilis, Mycoplasma pneumoniae, Sinorhizobiummeliloti, Vibrio cholerae, Chlamydia trachomatis, Pseudomonas syringae, and Staphylococcus aureus have been stated to have antisense RNA (asRNA) transcripts.^35^ The bacterial protein YidC is extremely preserved among pathogens and is necessary for membrane protein attachment and decrease of YidC production contributing to bacterial growth retardation. Therefore, it can be a novel potential target for therapeutic applications. Antisense RNA-mediated YidC down-expression in E. Colistrain resulted in identify antibacterial essential oils eugenol and carvacrol.^75^ The influence of the antisense oligomer is extremely particular to the targeted gene’s sequence, which is preserved in numerous bacterial types, and it does not have any noticeable toxicity against human cells.^45^ The combined CRISPR and asRNA system, can be applied to reversibly repress or derepress multiple target genes concurrently, permitting for rational reprogramming of cellular functions. Gene target are repressed/derepressed by CRISPR system from Streptococcus pyogenes and synthetic antisense RNAs (asRNAs) in Escherichia coli strains. In fact, when the CRISPR system represses the target gene, it can be derepressed by expressing asRNA which hide away a small guide RNA (sgRNA). In addition, up to 95% of derepression can be attained through designing as RNAs which target various regions of a sgRNA and by changing the hybridization free energy of the sgRNA–asRNA complex.^47^‏ RNA interference has been suggested to be an alternative method to prevent bacterial growth or demolish them. For this aim we suggest antisense therapy against type IV secretion system and Lep proteins synthesis in Legionella.

## Dendrimers-based antisense delivery system approaches for treatment of Legionellosis

The important therapeutic aim in biotechnology is the capacity to safely and professionally transfer external DNA, RNA, antisense or drug^76^ into cells.^63,77^ The capability to deliver fragments of DNA or RNA to the required section of a cell is a challenging issue. Substrate-mediated transfection, which withstands the release of knocked DNA or vector/DNA complexes and provides cell growth, has been established to solve the problems associated with the extracellular obstacles in gene delivery system.^78^ Rapid transfection which can achieve by viral carrier, immunological and oncologic side effects connected to these vectors have remained as controversial issues. Nowadays, non-viral gene delivery system to transfer genetic material to targeted cells such as natural/synthetic molecules or physical forces are preferred methods. They have some benefits such as targeting capability, simplicity of fabrication, possibility for repeat administration and low immune rejection.^79^ There are different dendrimers such as peptide, and glycopeptide, has capability to bind bacterial polysaccharides representing interesting tools for both therapeutics and diagnostics applications. Nowadays, because of higher bacterial resistance for common antimicrobial drugs, discovery of new antibacterial medications and diagnostic tools are very important .^80^ It has been reported that acid-triethylene glycol (GATG) dendrimers is valuable and versatile platform to develop a novel antimicrobial materials targeting microbial viability and/or virulence.^81,82^ The anti-bacterial sequences (ABS) can be integrated into plasmids, viral, and other vectors and packaged in liposomes or cationic polymers such as polyethylenimine (PEL) to prevent or reduce the likelihood of infections leading to sepsis.^65^ Dendrimers as non-viral gene delivery tools which can be utilized to deliver sequence of DNA or RNA as oligonucleotides to the certain part of cells are challenging experiments and novel method.^83,84^ Dendrimers as Nano-sized synthetic polymers have positive charge with distinct, homogeneous, and monodisperse organization containing tree-like arms or branches.^85^ Dendrimers are suitable and safe for the successful application in biomedicine such as imaging, drug delivery, gene delivery and photodynamic therapy.^86^ The highest benefits are the progress in the antifungal properties and antibacterial action, for example decrease in toxicity, bioavailability, and target tissue which simplifies advanced therapeutic methods.^87,88^

Valuable effort is being performed to elucidate the techniques of using dendrimers for gene trafficking into the cells without any interference of damage to the cell’s DNA. It is important to maintain DNA activity in the course of dehydration so dendrimer/DNA complexes need to be encapsulated and compressed in a water resolvable polymers, subsequently they are deposited on or inserted in functional polymer films with a fast degradation rate to facilitate gene transfection. Based on this method, for substrate-mediated gene delivery, Polyamidoamine (PAMAM) dendrimer/DNA complexes have been applied to encapsulate functional biodegradable polymers. Many reports have revealed that the fast-degrading functional polymer with excessive potential for localized transfection is a good tool.^78,86,89,90^‏ Antisense has negative charge and conjugates with dendrimers as a positive charge polymer. We have established Epidermal growth factor receptor (EGFR) and c-Src antisense oligonucleotide encapsulated with PAMAM dendrimers in human colon cancer cell line and have showed its effects on signalling pathway.^63,68,91^ To confirm entry of antisense to the cell, fluorescent microscope and Fluorescence-activated cell sorting (FACS) analysis have been carried out and showed that Fluorescein isothiocyanate (FITS) are conjugated effectively to dendrimers. Our studies evaluated the antisense dendrimers mediated transfer into cells and showed the effective antisense entry inside the cell; however, the antisense alone is not able to enter the cells. As a result, dendrimers could be safe and suitable tool to antisense delivery system for *L. pneumophila* treatment. Therefore, antisense against the type IV secretion system and Lep protein synthesis in Legionella encapsulated with dendrimers could be a novel approaches in Legionnaires' disease.

## Conclusion

Vesicle trafficking pathway in L. *pneumophila* could be as a target for eliminating or disarming pathogens via antisense therapy. Antisense therapeutic application for bacterial protein synthesis has role in mediating the intracellular trafficking pathway to avoid phagosome and lysosome fusion in macrophages. Some of these proteins have been shown to participate in the trafficking of the Legionella phagosome. By reducing these proteins through antisense therapy, bacteria could not be able to hijack host vesicle trafficking pathway, therefore phagosome and lysosome fuse inside the cell and they are killed in lysosomes through digesting by the lysosomal enzymes. Nowadays, instead of proteins based targeting as potential drug action, drug companies and researchers are interested in utilizing different RNA species, DNA, new protein families or macromolecular complexes of these components to treat and eliminate antibiotics resistance pathogens.

## Acknowledgments

The author thanks to Prof. Jamie Rossjohn and Associate Prof. Travis Beddoe at Monash University, Melbourne, Australia, for their attitude regarding *L. pneumophila*.

## Ethical Issues

Not applicable.

## Conflict of Interest

The authors report no conflicts of interest.
